# Effects of Willow Bark (Salix Extract) on Pain and Stress Following Disbudding of Organic Dairy Calves

**DOI:** 10.3390/ani15040575

**Published:** 2025-02-17

**Authors:** Madison E. Bacon, Marcia I. Endres, Bradley J. Heins

**Affiliations:** 1 Department of Animal Science, University of Minnesota, Saint Paul, MN 55108, USA; bacon097@umn.edu (M.E.B.); miendres@umn.edu (M.I.E.); 2 West Central Research and Outreach Center, University of Minnesota, Morris, MN 56267, USA

**Keywords:** animal wellbeing, disbudding, organic, willow bark

## Abstract

Cautery disbudding is a painful procedure and major welfare concern in organic dairy production, which lacks effective and easily implemented methods of pain control. Willow bark (WB) has been used to treat chronic pain in humans, but there is no scientific evidence that WB has an analgesic effect in dairy cattle. The objective of this study was to evaluate the effect of an oral WB bolus on heart rate, salivary cortisol concentration, ocular temperature, and lying behavior (time, bouts, and bout duration) of 42 Holstein and crossbred calves. Heifer calves were born at the University of Minnesota West Central Research and Outreach Center, Morris, MN organic dairy. Calves were disbudded from 4 to 7 weeks of age (42 ± 3 d) and randomly assigned to one of three treatments (*n* = 14 calves/treatment): hot iron disbudding with lidocaine (LID; 5 mL per horn bud), hot iron disbudding with oral WB (200 mg/kg), or sham disbudding (SD). Saliva samples and thermal images were collected 1 h before disbudding, at disbudding, and 5 min, 10 min, and every 30 min until 240 min after disbudding. Cardiac monitors recorded heart rates continuously throughout the 5 h study period. HOBO loggers recorded lying behavior during the 72 h following disbudding. The variables included in the statistical model for analyses were fixed effects of treatment, time, and the interaction between treatment and time, and calf was a random effect. The mean heart rate of LID calves (123.3 ± 2.8 beats/minute) and WB calves (124.5 ± 3.2 beats/minute) were higher than SD calves (110.8 ± 2.3 beats/minute). Salivary cortisol concentrations were higher for the LID calves (103.8 pg/mL) and for the WB calves (103.4 pg/mL) than for the SD calves (85.5 pg/mL). The ocular temperature was not affected by treatment group. There were fewer lying bouts 24 to 48 h after disbudding compared to the first 24 h after disbudding in both the LID and WB treatment groups. Treatment group did not affect lying time or lying bout duration. Neither LID nor WB alleviated the pain of calves throughout the post-disbudding period. The results highlighted the need to discover suitable options for pain mitigation of calves in organic livestock production.

## Simple Summary

Cautery disbudding in organic dairy farming currently lacks effective alternative methods for pain management. This study evaluated the analgesic effects of willow bark on disbudded Holstein and crossbred heifer calves. Heart rate and salivary cortisol were higher in lidocaine and willow bark calves compared to sham calves. Lying bouts were less frequent 24 to 48 h after disbudding in lidocaine and willow bark calves compared to the first 24 h after disbudding. The findings underscore the need for viable pain control options for disbudding in organic livestock production.

## 1. Introduction

Disbudding, a common husbandry procedure on dairy farms [1], is considered a major animal welfare concern within the dairy industry [2]. Cautery disbudding, which utilizes a hot iron to prevent horn growth by cauterizing the horn buds of young dairy calves, is painful [3]. Pain and stress in cattle is often quantified through physiological measures such as change in heart rate [4], ocular temperature [5], and cortisol concentrations [6], as well as behavioral measures such as lying time [7].

Administration of a local anesthetic, such as lidocaine, mitigates acute pain during the disbudding procedure [6,8]. A non-steroidal anti-inflammatory drug (NSAID) may alleviate lingering pain following disbudding [9], which may last up to 24 h [10]. The National Dairy Farmers Assuring Responsible Management (FARM) Program requires participating producers, which includes 99% of the US dairy milk supply, to provide a form of pain control for disbudding [11]. For organic dairy production, only materials approved by the National Organic Program (NOP) may be provided for pain relief [12]. The use of pain mitigation for disbudding has increased in recent years [13], but the lack of effective options for alleviating disbudding pain remains a significant welfare issue for organic dairy producers. Lidocaine is approved by the NOP for use in organic livestock production [12] and is commonly used to control pain during the disbudding procedure. Multimodal therapies which combine a local anesthetic and an NSAID, although preferable, are comparably rare [14,15].

Animal welfare can be conceptualized as three overlapping “spheres” representing the animal’s biological functioning, natural living, and affective state [16]. Organic livestock producers often value natural living above biological functioning or affective state [17]; however, the hesitancy to use NOP-approved synthetic products for pain control is a common criticism of organic livestock production. A survey of organic dairy farms in the US found that only 26% of farms used either a local anesthetic, NSAID, or sedative for disbudding pain [18]. Organic farmers may oppose the use of synthetic substances such as lidocaine and flunixin meglumine in favor of herbal remedies, which more closely align with organic values. Organic producers have shown interest in homeopathy and natural pain remedies [18,19]. However, the lack of scientific evidence for the efficacy of natural or plant-based remedies in livestock is a major concern of critics of organic livestock production [17,18,20]. There is an urgent need to identify safe and effective alternative pain mitigation therapies for the disbudding of dairy calves [21].

Willow bark (Salix) has been used by humans for thousands of years and may alleviate chronic pain [22,23]. Salicin, the active ingredient in willows and the basis of aspirin [24], metabolizes into salicylic acid, which inhibits inflammation [25]. Synthetic salicylates have been used to alleviate pain associated with castration and dehorning in cattle [26,27]. Several products containing willow bark are marketed for controlling pain, although an effective dosage has not been determined [28]. No studies have evaluated the analgesic effects of willow bark on the disbudding of dairy calves.

The hypothesis of this study was that disbudded calves that received either a local anesthetic or an oral plant-based bolus would differ for physiological and behavioral pain responses following cautery disbudding compared with calves that were sham disbudded. Therefore, the objective of this study was to evaluate heart rate, ocular temperature, salivary cortisol concentration, and lying behavior of dairy calves disbudded after a local anesthetic or an oral willow bark bolus compared with calves that were sham disbudded.

## 2. Materials and Methods

### 2.1. Animals and Housing

The University of Minnesota Institutional Animal Care and Use Committee approved all experimental procedures (protocol number #2007-38250A). Calves were born from March to May 2021 at the University of Minnesota West Central Research and Outreach Center in Morris, MN, USA. The 42 pre-weaned female calves were in the age range from 33 to 52 d (mean ± standard deviation = 42 ± 4 d) at disbudding. Calves were either purebred Holstein (*n* = 6) or crossbred (*n* = 36) as described by Pereira et al. [29]. This experiment was performed as a generalized randomized complete block design. The sample size for this experiment was determined by methods described in the work of Phillips and Heins [30]. The estimated sample sizes needed to achieve a power > 0.80 for cortisol after disbudding were 8 calves per group. The maximum required sample size from these calculations was inflated by 40% to account for any potential dropped calves (8 × 1.40 = 11). Forty-two calves were used for this experiment.

Calves were housed in individual (*n* = 13) pens, in pairs (*n* = 12), or in superhutches with groups of 6 calves (*n* = 17). Calves were housed in individual plastic hutches (Calf-Tel Deluxe II; Hampel Corp., Germantown, WI, USA) that were 1.8 m long × 1.1 m wide × 1.2 m high with an attached fenced pen that was 1.8 m long × 1.2 m wide × 1.1 m high. Pair-housed calves were housed in 2 plastic hutches. The superhutches for group-raised calves included an indoor area (3.66 × 6.10 m) and an outside access space that measured 3.66 × 6.10 m. Individual- and pair-housed calves were fed 10 L organic whole milk per calf twice daily with a 6 L Peach Teat Single Calf Feeder (Skellerup Industries Limited, Christchurch, New Zealand). Calves were offered 10 L of milk per day divided into 2 feedings. Individual intakes were not measured. Group-housed calves were fed 10 L milk per calf twice per day from an 80 L Calfateria Peach Teat Feeder (Stallion, Palmerston North, New Zealand). Prior to disbudding, calves were acclimated to human handling through a weekly recording of body weight.

### 2.2. Treatments

Calves were assigned to one of three treatments (*n* = 14 calves/treatment): a corneal nerve block via local anesthetic lidocaine injection prior to cautery disbudding (LID), an oral willow bark bolus (Nature’s Way, Green Bay, WI, USA) administered prior to cautery disbudding (WB), and sham disbudding (SD). Each bolus contained 400 mg of willow bark within a plant-based capsule. Treatments were balanced by breed and calf housing system. Disbudding occurred in blocks of 6 to 9 calves based on birth order and disbudding treatments were replicated across time periods from 28 April and 30 June 2021. A disbudded group without pain relief was not allowed according to the University of Minnesota Institutional Animal Care and Use Committee.

On the day of disbudding, all calves were restrained in a mobile chute with a headlock (Weighing Caf-Cart^®^, Raytec LLC, Ephrata, PA, USA). Calves in the LID group received 5 mL of 2% lidocaine per horn bud 5 min before disbudding. Calves in the WB group received a willow bark bolus at 200 mg/kg of the calf’s bodyweight, administered orally 20 min before disbudding. A WCROC study was unable to determine an effective dose of oral white willow bark [28]; therefore, this dose was based on prior studies utilizing similar products (sodium salicylate, ref. [31]; aspirin, ref. [32]). A heated cautery iron (Express Pistol-Grip Dehorner, Coburn, Whitewater, WI, USA) was applied to each horn bud. For sham-disbudded calves, the iron was unheated; however, calves were handled in a similar manner as calves that were disbudded to account for stress related to handling [30]. One single trained person administered the bolus and lidocaine and disbudded the calves throughout the experiment. All calves were disbudded 39 min apart between 0800 and 1233 h. This interval allowed for a minimum of 2 min between sample collection.

### 2.3. Data Collection

#### 2.3.1. Heart Rate

Calves were fitted with a Polar cardiac belt attached to extra-large chest straps (Polar H10, Polar Electro Ol, Kempele, Finland). The straps were tightened, and the clasps were wrapped with SyrFlex cohesive bandages (SyrVet Canada, St-Alphonse-de-Granby, QC, Canada) and duct tape to prevent the strap from falling off or from being chewed off by other calves. One researcher applied a general lubricant to the chest, sides of the calf, and under the front legs of each calf to moisten the electrodes per the instructions of use for the Polar H10 device. Previous studies that utilized cardiac monitors for calves and cows have recommended to provide time to habituate to the device [33,34], and therefore, the devices were attached 30 min prior to baseline readings. Observations of behavior at the time and visual inspection of the HR data during this period suggest that this amount of time was sufficient for habituating the animals to the devices.

Heart rate was measured continuously from 1 h before disbudding through 4 h post-disbudding with the Polar app on a Samsung Galaxy Tab A6 tablet (Samsung Electronics, Ridgefield Park, NJ, USA) connected via Bluetooth to each cardiac device. The tablets were placed near the calf hutch to ensure continued connectivity with the cardiac belt. After 4 h, the heart rate sessions were synced to the Polar desktop app and exported as csv files and organized into 1 min intervals with Microsoft Excel (Microsoft Corp., Redmond, WA, USA). A pre-disbudding baseline value was calculated by removing statistical outliers and averaging the beats per minute (bpm) of the first hour of recording. Outliers were defined as observations that deviated more than 1.5 times the size of a calf’s interquartile range of heart rate during the hour prior to disbudding. Heart rate readings failed to sync properly for 2 calves.

#### 2.3.2. Ocular Temperature

One researcher photographed the left eye of each dairy calf 1 h prior to disbudding, immediately before and immediately after disbudding, and at 5, 10, 30, 60, 90, 120, 150, 180, 210, and 240 min post-disbudding. A handheld calibrated thermal camera (Flir E60, FLIR Systems AB, Danderyd, Sweden) photographed the eye from approximately 0.5 m. Images were recorded with the FOL 18 mm lens and were 320 × 240 pixels in resolution. Images were analyzed with FLIR Tools software version 6.4. For each image, the emissivity was 0.98 and the atmospheric temperature and relative humidity were input into the software to account for changing climactic conditions. The atmospheric temperature and vapor pressure of the air were collected for each study period from the ATMOS 41 All-in-One Weather station (METER, Pullman, WA, USA) located at the West Central Research and Outreach Center. The elliptical tool encompassed the entire eye plus an approximately 1 cm margin around the eye. The maximum temperature for the entire eye was manually recorded in Microsoft Excel.

#### 2.3.3. Salivary Cortisol Concentration

Saliva samples were collected from each calf in the home pen 1 h prior to disbudding, immediately before and immediately after disbudding, and at 5, 10, 30, 60, 90, 120, 150, 180, 210, and 240 min post-disbudding. A cotton swab (Salivette, Sarstedt, Aktieng-esellschaft and Co. Nümbrecht, Germany) was placed into the calf’s mouth with forceps and the calf chewed until the swab was soaked with saliva. If attempts to collect a sample without restraint were unsuccessful, a second handler held the calf using gentle handling. Swabs were placed in tubes and immediately stored on ice until centrifuged at 1750× *g* for 5 min. Samples were stored at −80 °C and shipped on dry ice to the Iowa State Veterinary Diagnostic Laboratory (Ames, IA, USA) for analysis. Eight samples (1.59%) did not have enough saliva for analysis.

#### 2.3.4. Lying Behavior

All calves had an electronic data logger (Hobo Pendant G, Onset Computer Corp., Bourne, MA, USA) to record lying behavior. The loggers were wrapped with SyrFlex cohesive bandages to provide cushion and labeled to ensure correct orientation and attached with SyrFlex cohesive bandage to the lateral side of the right hind leg above the metacarpophalangeal joint [35]. HOBO Suite software (Version 3.7.25) programmed the loggers to record the g-force and tilt and of the x-, y-, and z-axes at 60 s intervals [36]. Lying time, lying bouts, and lying bout duration were recorded through the 3 days following disbudding. The intervals recorded prior to disbudding were removed. Data were summarized in 24 h periods following disbudding for each calf. Due to a technical failure of the HOBO logger, 1 calf was removed.

#### 2.3.5. Statistical Analyses

All statistical analyses were performed with PROC MIXED of SAS 9.4 [37]. Pre-disbudding baselines were included as a covariate for heart rate, ocular temperature, and salivary cortisol. Normality was assessed visually with histograms. For the analysis of salivary cortisol, ocular temperature, and mean lying bout duration, outcomes were transformed to the natural log [38].

All statistical models included the fixed effects of treatment, time, the interaction of treatment and time, and the baseline values. The Akaike Information Criterion goodness of fit test determined additional covariates for the final models. The model for heart rate included the covariates of age and breed, with calf as a random effect. The model for ocular temperature included the covariates of age, breed, housing type, and air temperature, and the interactions of housing type, treatment, and time and air temperature, treatment, and time. Pearson correlation determined the association of ocular temperature and air temperature.

For analysis of lying behavior (daily lying time, lying bouts per day, and mean lying bout duration), statistical models included the fixed effects of treatment, day, and the interaction of treatment and day. Calf was included as a random effect. The model for lying time included the covariates of housing type, age, and breed, and the interactions of treatment, and housing type, day and housing type, and treatment and day and housing type. The MIXED procedure of SAS was used for daily lying time and daily mean lying bout duration. The frequency of lying bouts per day was first assessed for overdispersion (χ^2^/df = 0.68) and the Poisson regression model was used with the GLIMMIX procedure of SAS with housing type and age as covariates [37].

For ocular temperature and cortisol concentrations, back-transformed means are re-ported with back-transformed 80% confidence intervals and standard errors. For main effects, the Tukey adjustment was applied for pairwise comparisons. All treatment results were reported as least squares means and significance declared at *p* < 0.05.

## 3. Results

### 3.1. Heart Rate

**Figure 1:**
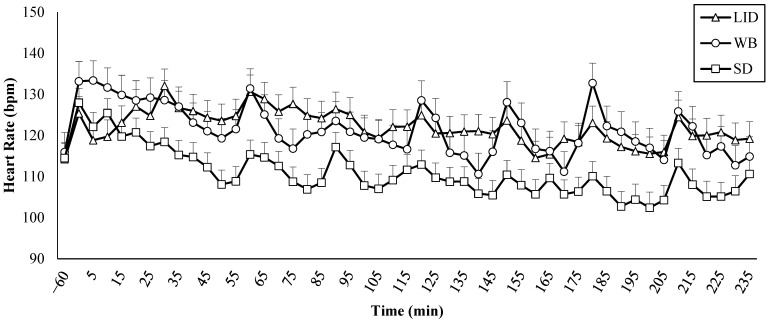
Least squares means and standard errors of mean heart rate of disbudded calves (*n* = 40) in 5 min intervals. LID = disbudded with lidocaine; WB = disbudded with oral willow bark bolus; and SD = sham disbudded. Disbudding occurred at 0 min.

All treatment groups had similar baseline mean heart rate (LID = 113.9 ± 3.9 bpm; WB = 121.0 ± 4.3 bpm; SD = 113.8 ± 3.9 bpm; *p* = 0.38). The effects of treatment, time, the treatment by time interaction, and baseline mean heart rate all significantly explained variation in heart rate (*p* < 0.001). The effects of age, housing type, and breed were not significant (*p* > 0.28). The mean heart rate of LID calves (123.3 ± 2.8 bpm) was higher (*p* < 0.001) than SD calves (110.8 ± 2.3 bpm), and the mean heart rate of WB calves (124.5 ± 3.2 bpm) was higher (*p* < 0.001) than SD calves (Figure 1). The LID and WB calves had similar mean heart rates (*p* = 0.926). Mean heart rates across treatment groups were 13.9 ± 2.3 bpm higher than baseline during the 5 min interval immediately following disbudding (*p* < 0.001) and remained above baseline in the following intervals after disbudding: 5 to 10 min (9.9 ± 2.3 bpm, *p* = 0.015), 10 to 15 min (10.7 ± 2.3 bpm, *p* = 0.003), 15 to 20 min (9.4 ± 2.3 bpm, *p* = 0.04), 20 to 25 min (10.6 ± 2.3 bpm, *p* = 0.01), 30 to 35 min (11.5 ± 2.3 bpm, *p* < 0.001), and 60 to 65 min (10.9 ± 2.3 bpm, *p* = 0.002). Within the LID calves, heart rates were 17.9 bpm higher (*p* = 0.03) 90 min after disbudding than at baseline. The heart rates of WB were 25.7 bpm higher (*p* = 0.03) 240 min after disbudding than the SD calves. All other pairwise comparisons of the interaction of treatment by time did not significantly differ (*p* > 0.11). The mean heart rate at the time of disbudding (0 to 5 min) was 126.6 ± 3.9 bpm for the LID calves, 136.2 ± 4.3 bpm for the WB calves, and 127.9 ± 3.5 bpm for the SD calves. Heart rate peaked for the LID calves at 133.4 ± 3.9 bpm at 30 to 35 min after disbudding, for the WB calves at 136.4 ± 4.3 bpm at 5 to 10 min after disbudding, and for the SD calves at 0 to 5 min after disbudding.

### 3.2. Ocular Temperature

**Figure 2:**
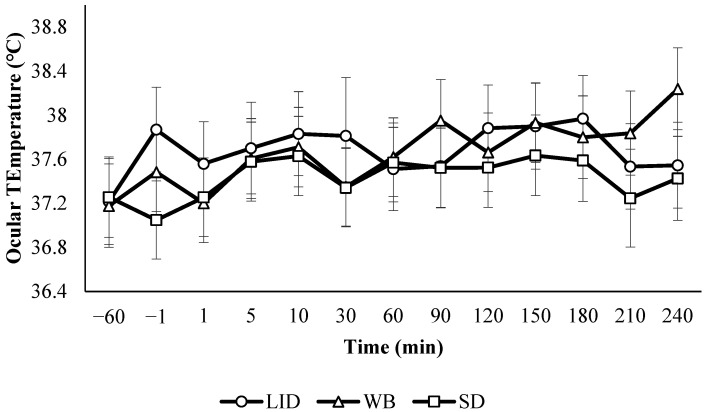
Least squares means and 80% CIs for the effect of treatment and sampling time on ocular temperature (*n* = 41). LID = lidocaine; WB = oral willow bark bolus; and SD = sham disbudding.

**Figure 3:**
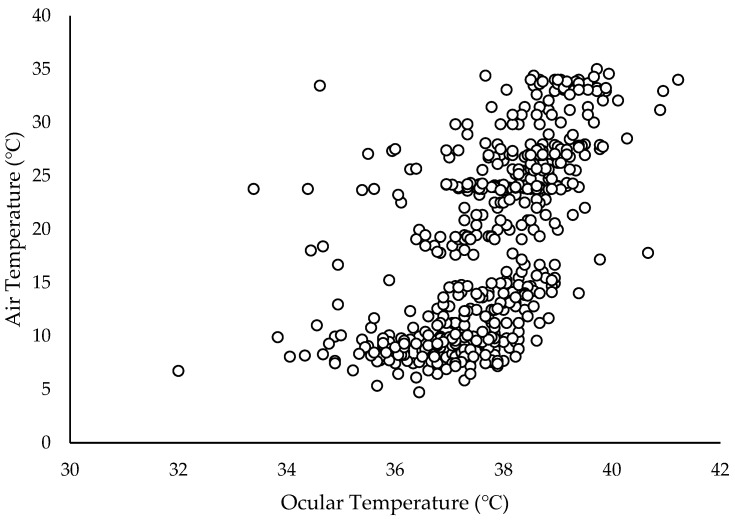
Ocular temperature (°C) by air temperature (°C). Pearson’s rank correlation identified a moderate positive correlation (R = 0.596, *p* < 0.001).

All calves had similar ocular temperatures at the baseline (*p* = 0.77). The effects of treatment, time, air temperature, and housing type significantly explained variation in ocular temperature (*p* < 0.05). The effects of the log of baseline ocular temperature, breed, age, and the interactions between treatment and time, housing type and treatment and time, and air temperature and treatment and time were not significant (*p* > 0.14). There were no differences between treatments or sampling times (Figure 2). Ocular temperature was lower (*p* = 0.01) for individually housed calves (37.3 °C) than for pair-housed calves (37.9 °C). The Pearson correlation showed a moderate positive correlation between the natural log of ocular temperature and air temperature (R = 0.60, *p* < 0.001; Figure 3).

### 3.3. Salivary Cortisol

Treatment and time explained (*p* < 0.01) variation in salivary cortisol. All treatment groups had similar baseline salivary cortisol concentrations (*p* = 0.80). Across time points, salivary cortisol concentrations were higher (*p* = 0.03) for the WB calves (103.4 pg/mL) than the SD calves (85.5 pg/mL) and higher (*p* = 0.02) in the LID calves (103.8 pg/mL) than the SD calves. The LID and WB calves were not different for salivary cortisol (*p* = 0.99). Across treatments, salivary cortisol was higher (*p* = 0.004) 30 min after disbudding (115.3 pg/mL) compared to baseline (83.0 pg/mL).

**Figure 4:**
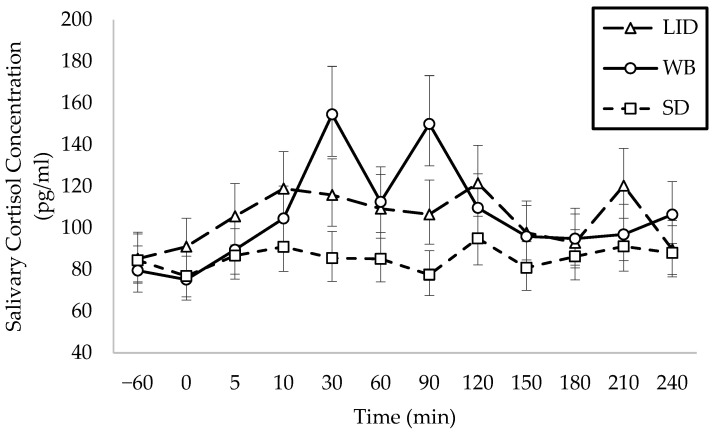
Least squares means and 80% CIs for the effect of interaction of treatment and sampling time on salivary cortisol concentration (*n* = 42). LID = lidocaine; WB = oral willow bark bolus; and SD = sham disbudding. Disbudding occurred at 0 min.

Salivary cortisol concentrations were higher (*p* < 0.01) for the WB calves (150.0 pg/mL than the SD calves (77.5 pg/mL) at 90 min (Figure 4). Within the WB calves, cortisol was above the baseline at 30 (154.6 pg/mL *p* = 0.002) and 90 (150.0 pg/mL; *p* = 0.01) min after disbudding. Cortisol peaked in the WB calves at 30 min after disbudding; by comparison, the LID and SD calves peaked at 120 min with cortisol concentrations of 120.3 pg/mL and 95.0 pg/mL.

### 3.4. Lying Behavior

For lying time, treatment, day, and the interaction of treatment and day (Table 1) were not significant (*p* > 0.31). Housing type influenced lying time (*p* < 0.001), and the interactions of housing type, treatment, and day were not significant (*p* = 0.55). Daily lying time was lower in group-housed calves (1013.0 ± 14.7 min) than in individually housed calves (1074.2 ± 15.4 min; *p* = 0.01) and lower in paired calves (936.5 ± 17.8 min) than individually housed calves (*p* < 0.001).

**Figure 5:**
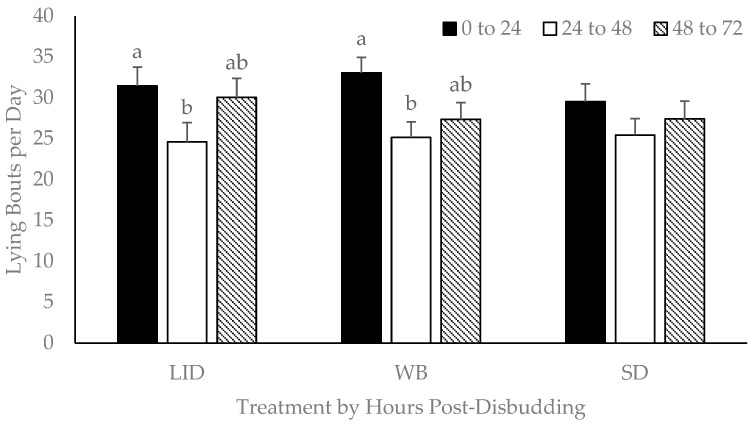
Least squares means and standard errors for the effect of treatment and sampling time on frequency of lying bouts per day (*n* = 41). LID = lidocaine; WB = oral willow bark bolus; and SD = sham disbudding. Labeled means without a common letter within treatment differ (*p* < 0.05).

Daily frequency of lying bouts was affected by day (*p* < 0.001) and housing type (*p* = 0.03), while treatment and treatment by day interaction were not significant (*p* > 0.42). Across treatments, the mean number of lying bouts in the first 24 h following disbudding was 31.3 ± 1.3 bouts, which was higher than both the 24-to-48 h period following disbudding (25.1 ± 1.1 bouts; *p* < 0.001) and the 48-to-72 h period following disbudding (28.2 ± 1.2; *p* = 0.01). The 24-to-48 h period after disbudding had fewer lying bouts than the 48-to-72 h period (*p* = 0.02). Within the LID treatment, lying bouts decreased (*p* = 0.03) from 31.5 ± 2.3 in the first 24 h after disbudding to 24.6 ± 1.9 bouts during the 24-to-48 h period after disbudding (Figure 5). Within the WB treatment, lying bouts decreased (*p* = 0.01) from 33.0 ± 2.4 during the first 24 h after disbudding to 25.1 ± 1.9 bouts during the 24-to-48 h period after disbudding. The mean number of lying bouts per day in group-housed calves (24.6 ± 1.3 bouts) was lower (*p* = 0.03) than in paired calves (31.1 ± 2.2 bouts).

For the daily mean lying bout duration, the effects of treatment and the interaction of treatment and day (Table 2) were not significant. Day (*p* = 0.001) and housing type (*p* = 0.01) had an effect on lying bout duration. The mean lying duration in the first 24 h (34.6 min) after disbudding was lower (*p* < 0.001) than in the 24-to-48 h after disbudding (40.6 min). The mean lying duration in group-housed calves (42.7 min) was higher (*p* = 0.003) than in paired calves (32.23 min).

## 4. Discussion

Though not statistically significant, WB calves had the highest mean heart rate immediately after disbudding, followed by LID calves, while SD calves had the smallest increase in heart rate during this period. These results were consistent with studies that showed that the use of a local anesthetic reduced the immediate cardiac response following disbudding [5,39]. The heart rate did not differ between the LID and WB calves; however, the LID and WB calves had higher heart rates than SD calves. Higher heart rates in disbudded calves compared to sham calves was also reported by Heinrich et al. [39]. All treatment groups had several spikes in heart rate during sampling times at 5, 10, and every 30 min through 240 min (Figure 1). Dairy calves tend to avoid human handlers [40], which indicates that handling is a stressful event. Therefore, the process of handling calves for sampling in the current study may have been a stressor for the calves and potentially affected cardiac responses.

Ocular temperature followed the trend reported by Stewart et al. [5], where calves decreased in temperature immediately after disbudding followed by an increase in temperature over the next several minutes (Figure 2). However, the changes observed in this study were less dramatic than those noted by Stewart et al. [5] and were not significant. A non-significant decrease in temperature occurred in LID calves between 180 and 210 min post-disbudding. A similar, significant decrease in ocular temperature between 2 and 3 h after disbudding was reported by Stewart et al. [41], where this pattern was attributed to the return of sensation to the horn buds. However, there was also a moderate positive correlation between air temperature and ocular temperature in the current study (Figure 3). Air temperatures during this study ranged from 4.7 °C to 35.1 °C and the widest range of temperatures observed in a single day was a 24.6 °C difference between the beginning and the end of the study period. The calves at the WCROC are housed outdoors, rendering it impossible to control atmospheric conditions over the 5 h period of this study. Ocular temperature may be more useful as a measure of stress for a shorter window of time after the stressor or when air temperature is held constant over long periods. The effect of housing type on ocular temperature was unexpected. Calves in paired housing spend much of their time lying together in the same hutch [42]. Quite possibly, the paired calves shared more body heat due to these social interactions and ocular temperature is correlated with core body temperature [43]. Therefore, this may explain lower ocular temperature in individual calves. Future studies should take calf housing into consideration if ocular temperature is used as a measure of stress.

Calves disbudded with the salix oral bolus had the highest salivary cortisol response, followed by local anesthesia. The result was similar to those reported by Phillips and Heins [30], where the cortisol response of calves that received an experimental herbal tincture was higher than the cortisol response in calves that received lidocaine. The cortisol response of calves that received the tincture in that study peaked at 30 min, which was similar to the WB calves in the current study. Graf and Senn [44] reported a similar delayed peak in cortisol concentrations of calves disbudded without local anesthesia. Salivary cortisol of WB calves differed from the baseline cortisol concentration at 30 and 90 min after disbudding and differed from SD calves at 90 min. The absence of a comparable change in cortisol concentration observed in either the LID or SD treatments, the latter of which was included to control for handling stress, strongly suggests that handling was not a significant factor contributing to the elevated cortisol levels noted in the WB treatment.

Calves that received local anesthesia peaked at 10 min after disbudding with a second non-significant rise in cortisol at 150 min [44]. In the current study, the LID calves saw a similar non-significant peak in cortisol at 120 min after disbudding. The functional duration of lidocaine is approximately 90 min [45], and therefore, the peak may have indicated the return of sensation to the horn buds.

Salivary cortisol concentration is generally considered a minimally invasive measure of stress while strongly correlated with plasma cortisol [46]. However, there may be a time lag between the stressor and peak salivary cortisol concentrations [47], which has been inconsistently reported. Delays in peak cortisol concentrations between saliva and plasma cortisol have been noted in humans (5.5 to 7.5 min; [48]) and sheep (20 to 30 min; [46]). Some studies have indicated that no time lag exists between plasma and saliva cortisol concentrations in cattle [49,50]. However, Hernandez et al. [47] found that salivary cortisol concentrations peaked at a 10 min lag behind plasma cortisol.

In the current study, there were no significant differences in lying time, number of lying bouts, or lying bout duration between treatments. It is important to note that there was no baseline lying behavior to compare to in the current study, and therefore, changes in behavior within calves from before and after disbudding could not be accounted for. However, comparisons between both disbudded calves and the control SD calves had no significant differences over the 72 h after disbudding. These results contradict the findings of other studies that have reported increased transitions from lying to standing [51] and less lying time after disbudding [52,53]. However, Doherty et al. [8] also reported no significant differences in lying times of calves dehorned with lidocaine, with saline, or not dehorned. The LID and WB calves had a numerical decrease in the number of lying bouts from the first 24 h after disbudding to the 24-to-48-hour period after disbudding. A decrease in lying bouts may indicate discomfort in calves [54]. The type of calf housing affected all lying behaviors assessed in this study. Mahendran et al. [55] reported no differences among lying times of individual and paired calves, but paired calves were more active than individual calves. Disbudded calves in group housing tended to have lower lying time than non-disbudded calves [7]. Calf housing is considered an important topic in dairy cattle welfare; future research could delve deeper into the question of how different rearing systems may impact welfare around disbudding.

This study was somewhat limited by a smaller calving season, which reduced the number of calves available for enrollment. Although an additional treatment group evaluating the NOP-approved NSAID, flunixin meglumine, would have been ideal, it was not possible with the number of calves expected during the calving season.

The results of the current study supported the hypothesis that the physiological pain and stress responses of calves differed by treatment. Heart rates and salivary cortisol concentrations were higher in both LID and WB calves than in SD calves. Lying behavior did not differ by treatment. Furthermore, in all of the behavioral measurements reported in the current study, the LID and WB calves did not differ from each other.

It is well established that non-steroidal anti-inflammatory drugs (NSAIDs) alone are ineffective at alleviating acute pain from cautery disbudding [56]. Pain from the disbudding procedure may last up to 24 h [10], which necessitates effective pain management. Although LID and WB calves did not differ across the behavioral measurements assessed in this study, the lack of significant physiological changes at the time of the disbudding procedure indicated that lidocaine successfully desensitized the horn buds to acute pain. Furthermore, cortisol concentrations were highest in WB calves, which suggests these calves experienced the strongest stress response. The American Veterinary Medical Association (AVMA) officially recommends a combination of local anesthetics, analgesics, and sedation for controlling disbudding pain [57]. Subdermal flunixin remains the only effective NSAID [58] approved by the USDA National Organic Program for use in organic production [12]. Therefore, a National Organic Program-approved multimodal pain management plan may include the combination of lidocaine as a local anesthetic and flunixin as an NSAID; however, opposition to synthetic substances in organic production remains a significant barrier. An effective herbal analgesic in combination with lidocaine as a local anesthetic may represent a possible compromise with significant improvements for organic dairy cattle welfare. Future research should prioritize the search for effective, easy-to-use pain remedies suitable for use in organic livestock production.

## 5. Conclusions

The local anesthetic lidocaine reduced acute pain in calves during disbudding as indicated by a lack of significant changes in heart rate, salivary cortisol concentration, or ocular temperature at the time of disbudding. Willow bark, which has not been approved by the FDA, did not appear to mitigate pain during disbudding. Neither lidocaine nor willow bark were effective at managing long-term pain after disbudding. At a minimum, organic dairy producers should provide lidocaine to alleviate acute disbudding pain. Further research is needed to identify effective organic options for relieving long-term inflammation following disbudding.

## Figures and Tables

**Table 1:** Least square means and standard errors of means for daily lying time (min) of calves by disbudding treatment and hours post-disbudding.

Hours Post-Disbudding	Treatment ^1^		
	LID	WB	SD
0 to 24 h	1030.44 ± 20.02	1016.36 ± 19.76	1053.82 ± 20.68
24 to 48 h	1002.44 ± 20.02	1021.19 ± 19.76	1026.17 ± 20.68
48 to 72 h	997.05 ± 20.02	1024.06 ± 19.76	1049.51 ± 20.68

^1^ Treatments: LID = cornual nerve block with 5 mL of 2% lidocaine per horn bud; WB = 200 mg/kg of oral willow bark bolus; SD = sham disbudding.

**Table 2:** Least square means and 80% confidence intervals for daily mean lying duration (min) of calves by disbudding treatment and hours post-disbudding.

Hours Post-Disbudding	Treatment ^1^		
	LID	WB	SD
0 to 24 h	33.8 (30.7 to 37.2)	33.2 (30.2 to 36.6)	36.9 (33.4 to 40.9)
24 to 48 h	41.4 (37.6 to 45.5)	41.2 (37.5 to 45.4)	39.3 (35.5 to 43.5)
48 to 72 h	35.2 (32.0 to 38.7)	37.7 (34.3 to 41.5)	38.4 (34.7 to 42.5)

^1^ Treatments: LID = cornual nerve block with 5 mL of 2% lidocaine per horn bud; WB = 200 mg/kg of oral willow bark bolus; SD = sham disbudding.

## Data Availability

Data available upon request from the corresponding author.
